# Perceived Stress, Cortical GABA, and Functional Connectivity Correlates: A Hypothesis-Generating Preliminary Study

**DOI:** 10.3389/fpsyt.2022.802449

**Published:** 2022-03-08

**Authors:** Jessica N. Busler, Eduardo Coello, Huijun Liao, Jacob Taylor, Wufan Zhao, Laura M. Holsen, Alexander P. Lin, Pamela B. Mahon

**Affiliations:** ^1^Department of Psychiatry, Brigham and Women's Hospital, Boston, MA, United States; ^2^Division of Women's Health, Department of Medicine, Brigham and Women's Hospital, Boston, MA, United States; ^3^Harvard Medical School, Boston, MA, United States; ^4^Department of Epidemiology, Harvard T. H. Chan School of Public Health, Boston, MA, United States; ^5^Department of Radiology, Brigham and Women's Hospital, Boston, MA, United States

**Keywords:** perceived stress, gamma-aminobutyric acid, magnetic resonance spectroscopy, prefrontal cortex, rfMRI

## Abstract

Stress exposures and dysregulated responses to stress are implicated in psychiatric disorders of mood, anxiety, and cognition. Perceived stress, an individual's appraisal of experienced stress and ability for coping, relates to dysregulated functioning in resting state brain networks. Alterations in GABAergic function may underlie perceived stress-related functional dysregulation in resting state networks but this has not yet been explored. Therefore, the current study examined the association of perceived stress, via the Perceived Stress Scale (PSS), with prefrontal GABA levels and corresponding resting state functional connectivity (RSFC) alterations. Twelve women and five men, ages 35–61, participated. MR spectroscopy was used to measure brain GABA levels in the anterior cingulate cortex (ACC), left dorsolateral prefrontal cortex (DLPFC), and ventromedial prefrontal cortex (VMPFC). Resting state functional scans acquired at 3 Tesla were used to measure RSFC within and between the default mode (DMN), salience (SN), and central executive networks (CEN), hippocampus and amygdala. We observed significant negative correlations between total PSS scores and left DLPFC GABA levels (*r* = −0.62, *p* = 0.023). However, PSS scores were not significantly correlated with RSFC measures (all *p* > 0.148). These preliminary results support a relationship between perceived stress and GABAergic functioning in DLPFC, a core node of the CEN, an intrinsic network thought to underlie goal-directed attentional processes. Our findings extend previous work suggesting that functioning in the CEN is related to perceived stress and may inform treatment strategies to improve outcomes in stress-related conditions.

## Introduction

Stress exposures are risk factors for psychiatric disorders including disorders of mood (1–3), anxiety (3) and cognition (4). Dysregulated psychological and physiological responses to stress are also common in these disorders. Psychological perceived stress is an individual's appraisal of experienced stress and ability for coping (5). Thus, perceived stress reflects the interaction between an individual and their environment and involves feelings of uncontrollability and unpredictability (6). Higher perceived stress is associated with adverse health outcomes including depression (7) and cognitive decline (8). However, neural mechanisms underlying interindividual differences in perceived stress are not fully known.

Resting state functional magnetic resonance imaging (rfMRI) is a method that involves observing spontaneous brain activity in the absence of a task and has been translated to investigate group differences in brain functioning, develop biomarkers of disease (9), and identify resting state networks (RSNs) with distinguishable brain functions (10). A triple network model involving aberrant activity among the default mode (DMN), salience (SN), and central executive networks (CEN) has been proposed to describe dysfunction in psychiatric disorders (11). The triple network model proposes that weak salience mapping by regions of the SN leads to aberrant engagement of the CEN and deficits in disengagement of the DMN. The DMN is believed to subserve self-referential mental activity such as emotion processing, rumination and thoughts of the past and future (12–15) and is primarily deactivated when attention is shifted to the external environment (16). The SN is thought to be involved in assigning salience to emotional and sensory stimuli (17) and to mediate the shift between the internally directed cognition of the DMN to more external cognitive processes (i.e. working memory and goal-directed cognition) characteristic of the central executive network (CEN) (18, 19). Dysregulated function within these three intrinsic networks has been linked to higher perceived stress with greater functional connectivity within the DMN (20), disrupted functional connectivity within the CEN (21), and greater functional connectivity between the SN and limbic regions (e.g. amygdala) (22). Perceived stress has been shown to be related to greater functional connectivity between the amygdala and the ACC and following a stress-reducing mindfulness intervention this functional connectivity between the amygdala and the ACC in individuals with higher perceived stress has been shown to decrease (22).

One potential mechanism underlying perceived stress and related functional dysregulation in resting state networks and limbic regions is altered GABAergic function. GABA is the primary inhibitory neurotransmitter in the brain and pre-clinical and clinical studies suggest that GABA modulates hypothalamic-pituitary-adrenal (HPA-axis) response to stress (23) which has been shown to be predicted, in part, by psychological perceived stress (24, 25). In addition, GABA has been shown to be decreased in the prefrontal cortex in depression (26) and increased in the dorsolateral prefrontal cortex (DLPFC) and ACC in Post-Traumatic Stress Disorder (PTSD) (27), two stress-related psychiatric disorders. The prefrontal cortex together with limbic structures (hippocampus and amygdala) is thought to form a regulatory network of the stress response by the HPA axis (28). However, the role of GABA in perceived stress and related network and limbic function has not yet been determined.

The current study aimed to preliminarily examine GABAergic functioning related to perceived stress and resting state functional connectivity (RSFC) with the goal of serving as a hypothesis generating study for future research investigating stress and GABAergic functioning. We employed proton magnetic resonance spectroscopy (^1^H-MRS), a non-invasive method for detecting, identifying, and quantifying bulk GABA in the human brain, at 7T where increased spectral dispersion and signal to noise provide more accurate measurements (29). We utilized the Perceived Stress Scale [PSS: (30)], a well-validated and psychometrically strong instrument for assessing perceived stress, and tested relationships between perceived stress and functional connectivity in resting state networks and limbic regions (e.g. DMN, CEN, SN, amygdala, and hippocampus) along with relationships between perceived stress and *in vivo* measurements of GABA levels in the VMPFC, DLPFC, and ACC, nodes of the DMN, CEN and SN, respectively.

## Methods and Materials

### Participants

Seventeen participants (12 women and 5 men) were recruited at Brigham and Women's Hospital through online advertisements targeted toward healthy participants and participants with mood disorder in order to enrich the sample for a range of emotion regulation in line with the NIMH Research Domain Criteria (31). Inclusion criteria were: (1) 35–64 years old, (2) with or without a research diagnosis of a mood disorder [lifetime bipolar I disorder, lifetime bipolar II disorder, recurrent major depression from MINI for DSM-IV (32)], (3) affectively stable, [i.e. absence of current depressed or manic mood episode from the Montgomery-Asberg Depression Rating Scale (MADRS) (33) and Young Mania Rating Scale (YMRS) (34)] and without current symptomatic posttraumatic stress disorder, (4) native English speaker able to read. Exclusion criteria were: (1) alcohol or substance abuse or dependence during the past 12 months, (2) positive toxicology screen, (3) dementia or mild cognitive impairment [from the Montreal Cognitive Assessment (MoCA) (35)], (4) contraindication to an MRI scan, (5) history of head injury resulting in loss of consciousness, (6) medical condition that would interfere with the MRI protocol, (7) current pregnancy.

### Assessment

After a phone screen, eligible participants were invited to participate in the study. Prior to the first study visit participants provided written informed consent. A psychiatrist or highly trained clinical MA-level coordinator administered the MINI to assess Diagnostic and Statistical Manual of Mental Disorders (DSM-IV) psychiatric research diagnoses. The MADRS and YMRS were administered to assess current depressive and manic symptoms. Participants additionally completed the Hopkins Adult Reading Test (36) as an indicator of full-scale IQ and the MoCA to assess possible dementia or mild cognitive impairment. Edinburgh Handedness Inventory (37) was used to assess handedness.

### Perceived Stress

Participants completed the PSS (30). The PSS is a 10 item self-report scale to measure individuals' perceptions of stress over the past month by assessing both experienced levels of stress and coping. The PSS has been validated in international samples (38–40) and has strong psychometric properties (41). Three participants did not complete the PSS and were not included in related analyses.

### ^1^H-MRS Acquisition and Processing

^1^H-MRS was performed on a clinical 7.0 Tesla MR scanner (Siemens Magnetom Terra) with a 32-channel receiver head coil. Prior to spectroscopy T1-weighted images were acquired with a 3D magnetization-prepared-rapid-gradient-echo (MPRAGE) sequence (TR = 2,290 ms, TE = 2.95 ms, voxel size = 0.7 × 0.7 × 0.7 mm^3^, acquisition matrix = 224 × 168, flip angle = 7°, slice thickness 0.70 mm, 240 total slices) and were used for localization of the voxel. Single-voxel MRS utilized a STEAM localization sequence (TR/TE/TM = 3,000/20 /10 ms, 128 averages) positioned in the dorsal anterior cingulate cortex (ACC, voxel size = 40 × 20 × 20 mm^3^), ventromedial prefrontal cortex (VMPFC, voxel size = 20 × 20 × 20 mm^3^), and left DLPFC (voxel size = 20 × 20 × 20 mm^3^) as shown in Supplementary Figure 1. Voxel locations were placed in three imaging planes by an experienced MRS technologist based on the following parameters. For ACC, the voxel is initially placed on a midline sagittal slice such that the anterior end of the voxel starts at the genu of the corpus callosum and the rotation of the voxel is such that it is parallel to the corpus callosum. Axial images are reviewed to ensure the voxel is midline and coronal images are reviewed to ensure that maximal gray matter is included. For VMPFC, the voxel is placed anterior to the genu of the corpus callosum such that the center of the voxel is along the bicommissural line. Axial and coronal images are reviewed to ensure that the voxel is in the midline and maximizes the amount of gray matter. For DLPFC, the voxel is placed on the coronal image between the superior and inferior frontal sulcus with a rotation that is parallel to the side of the skull. Axial and sagittal images are reviewed to ensure that the voxel does not contain the skull. Each voxel underwent automated optimization including 3-dimensional shimming, transmit gain, frequency adjustment, and water suppression. When the full width at half maximum (FWHM) of the water signal was ≥30 Hz, manual shimming was performed to optimize the magnetic field homogeneity of the selected spectroscopy volume of interest to a line width of <30 Hz FWHM of the absolute water signal.

OpenMRSLab was utilized for pre-processing including coil combination, frequency and phase correction as well as eddy current correction and residual water removal (42). LCModel software (43) was used for the ^1^H single voxel spectroscopy spectral fitting using simulated spectra of 19 metabolites as a customized basis set. All spectra were quality controlled by visual inspection of the spectra and according to the following criteria: (1) a FWHM linewidth of the unsuppressed water spectrum <30Hz, (2) signal-to-noise ratio (SNR) <5, and (3) Cramer-Rao Lower Bound (CRLB) of NAA >5. Spectra that did not meet these criteria were re-processed with eddy current and frequency shift correction. If issues remained after re-processing, those spectra were eliminated from further analyses. FWHM, SNR, and CRLB are described in Supplementary Table 3. GABA levels in institutional units (i.u.) were calculated in the LCModel output and standard correction for CSF was applied.

### rfMRI Acquisition and Processing

On the same day as the scan for ^1^H-MRS a second scan for rfMRI was performed on a clinical 3.0 Tesla MR scanner (Siemens TIM Skyra, VD13) with a 20-channel receiver head coil. Two participants did not complete this scan and were not included in related analyses. Whole-brain functional imaging was performed using a gradient-echo EPI pulse sequence with z-shim gradients to compensate for magnetic susceptibility-induced signal loss at the base of the brain (44–46) with the following parameters: 21 contiguous oblique-axial slices, 5.0 mm slice thickness, TR/TE = 1,200/30ms, flip angle = 90°, FOV = 240 × 240 mm, voxel size = 3.8 × 3.8 × 5.0 mm, 250 total images. Duration of the resting state scan was 5:06 min and participants were instructed to lie still with their eyes closed and let their mind freely wander. Participants communicated with study staff immediately before and after the scan to verify they were awake. One participant reported falling asleep during the scan and was excluded from related analyses. T1-weighted images were acquired with a 3D magnetization-prepared-rapid-gradient-echo (MPRAGE) sequence (TR = 2,300 ms, TE = 2.98 ms, voxel size = 1.0 × 1.0 × 1.1 mm, acquisition matrix = 248 × 256, flip angle = 9°, 176 sagittal slices) and used for co-registration.

We used the CONN Toolbox Version 19b (https://www.nitrc.org/projects/conn/) implemented through Matlab Version 2019b for processing structural and functional MRI data. We employed CONN's default preprocessing pipeline for volume-based analyses (direct normalization to MNI-space) which performs the following preprocessing steps via procedures in SPM12: functional realignment and unwarp; slice-timing correction; outlier identification; direct segmentation and normalization; and spatial smoothing. Specifically, functional data were coregistered and resampled to the first scan of each session using b-spline interpolation for the realignment and unwarping procedure. For slice-timing correction, functional data were time-shifted to correct for interleaved slice acquisition and resampled using sinc-interpolation to match the time in the middle of each acquisition time. Potential outlier scans were identified based on the observed global BOLD signal and the amount of subject-motion in the scanner using the “conservative” threshold settings in CONN of 0.5 mm for scan-to-scan frame-wise displacement and 3 s.d. away from the mean global brain activation across the entire volume for each timepoint. Functional and anatomical data were normalized into standard MNI space and segmented into gray matter, white matter, and CSF tissues classes with a resampling of functional data to 2 mm isotropic voxels and structural data to 1 mm isotropic voxels data using 4th order spline interpolation. Last, functional data were smoothed using a spatial convolution with a Gaussian kernel of 8 mm FWHM.

To account for artifact and physiological noise in the data we implemented CONN's default denoising pipeline involving linear regression of potential confounding effects in the BOLD signal and temporal filtering. The default denoising pipeline implements an anatomical component-based noise correction procedure (aCompCor) that includes noise components from cerebral white matter and cerebrospinal areas and includes other noise components of estimated subject-motion parameters (12 components), scrubbing of outlier scans (components vary per subject), and first order linear session effects. In addition, based on recommendations that higher frequencies contain informative resting state signals (47) and based on processing pipelines implemented in other studies including the Human Connectome Project (48–50) we implemented a temporal high-pass filter of 0.01 Hz to minimize the influence of physiological, head-motion, and other noise sources while focusing on frequency fluctuations informative of resting-state networks.

### Statistical Analysis

Relationships between continuous variables were tested using Pearson's correlations and *t-*tests were used to test relationships involving dichotomous variables. Given evidence of differential responses to stress by biological sex and age, we assessed these variables along with diagnosis, trauma exposure, symptom ratings, and handedness in relation to PSS scores.

#### MRS Analyses

We tested for correlations between continuous measures of GABA concentrations and total PSS scores using Pearson's correlations. In this hypothesis generating, preliminary analysis we did not correct for multiple testing. When a nominally significant correlation between PSS score and GABA was observed, the voxel was carried forward for functional connectivity analyses.

#### Functional Connectivity Analyses

Emergent nominally significant correlations between GABA and PSS scores were used to inform seed region selection for subsequent RSFC analyses. Masks of the MRS-determined voxel region were created for each subject and translated into MNI space for use as subject-specific seed ROIs in RSFC processing. We conducted seed-based correlations between the MRS-determined seed and network nodes in the DMN (posterior cingulate cortex [PCC], left and right lateral parietal [LP], medial prefrontal cortex [mPFC]), CEN (left and right lateral prefrontal cortex [LPFC] and left and right PPC), and SN (ACC, left and right insula, left and right supramarginal gyrus [SMG], left and right rostral prefrontal cortex [RPFC]) and with limbic areas (bilateral hippocampus, bilateral amygdala) using preconstructed ROIs from the Harvard-Oxford atlas in CONN. Network nodes were chosen based on predefined network assignment in CONN.

To examine functional connectivity between the seed region with each ROI we conducted ROI-to-ROI connectivity analyses which produce weighted connectivity matrices computed from a Weighted Least Squares (WLS) linear model to characterize functional connectivity strength. For second-level analyses CONN implements a General Linear Modeling (GLM) framework for testing between-subjects and between-conditions contrasts. We conducted a one-sample *t*-test to determine ROI-to-ROI connectivity from the seed region to each of our pre-defined ROIs. Results were thresholded at an analysis-level false discovery (FDR) rate of *p* < 0.05 to correct for the total number of connections tested from the seed ROI. Bivariate correlation coefficients were calculated between the average time-courses for the seed to each ROI in our pre-defined set and converted to Fisher-transformed *z*-scores. *Z*-scores were extracted for significant RSFC results from the ROI-to-ROI analyses for use in primary analyses where separate correlation analyses were used to test the relationship of RSFC results with PSS scores and with cortical GABA. Results were deemed significant at a nominal threshold of *p* < 0.05.

## Results

### Demographic and Clinical Characteristics

In total, of the 17 original subjects, 3 did not complete the PSS, 1 reported falling asleep during the 3T scanning session and was removed from related analyses, and in 3 participants GABA measurements did not meet our quality standards and were removed from their respective analyses (1 participant removed from DLPFC, 1 participant removed from ACC, and 1 participant removed from VMPFC analyses). Consequently, for comparisons involving perceived stress and GABA levels as well as comparisons involving perceived stress and RSFC there was complete data for 13 participants and for comparisons involving GABA levels and RSFC, 15 participants. In total 12 participants had complete data across all analyses. Demographic and clinical features of the participants are shown in Table 1. Three participants (17.6%) had an anxiety disorder. As expected, younger age (*r* = −0.61, *p* = 0.021) and mood disorder diagnosis (*t*_12_ = 3.68, *p* = 0.003) were significantly related to greater total PSS scores. None of the demographic or clinical variables tested were significantly associated with GABA levels (*p* > 0.054), but a trend was present for mood disorder diagnosis.

**Table 1:** Demographic, clinical characteristics, and relevant relationships with PSS scores and DLPFC GABA.

	***n =*** **17**	**PSS score (*n =* 14)**	**DLPFC GABA (*n =* 16)**
Age	47.2(8.2)	***r*** **=** **−0.61**, ***p*** **=** **0.021**	*r* = 0.27, *p* = 0.314
Sex (% female)	70.6%	*t*_12_ = 0.68, *p* = 0.506	*t*_14_ = −1.67, *p* = 0.117
Handedness (% RH)	76.5%	*t*_12_ = 0.44, *p* = 0.671	*t*_14_ = −0.54, *p* = 0.598
Mood Diagnosis (% yes)	47.1%	***t*****_12_** **=** **3.68**, ***p*** **=** **0.003**	*t*_14_ = −2.10, *p* = 0.054
Trauma Experience (% yes)	35.3%	*t*_12_ = 0.25, *p* = 0.810	*t*_14_ = −1.12, *p* = 0.282
MADRS	2.3(6.0)	*r* = −0.04, *p* = 0.890	*r* = 0.23, *p* = 0.420
YMRS	1.7(2.5)	*r* = −0.10, *p* = 0.745	*r* = 0.13, *p* = 0.653
PSS	43.2(9.6)	N/A	***r*** **=** **−0.62**, ***p*** **=** **0.023**
DLPFC GABA (i.u.)	1.0(0.3)	***r*** **=** **−0.62**, ***p*** **=** **0.023**	N/A

*Results presented as mean (SD) or %. PSS, Perceived Stress Scale; MADRS, Montgomery-Asberg Depression Rating Scale; YMRS, Young Mania Rating Scale; RH, Right Hand; DLPFC GABA, DorsoLateral PreFrontal Cortex Gamma-Amino butyric Acid; i.u., institutional units. For dichotomous variables the group shown represents the comparison group and the group not shown represents the reference group for statistical testing. Bold values indicate statistically significant results at a significance threshold of p < 0.05*.

### Perceived Stress and GABA Levels

**Figure 1:**
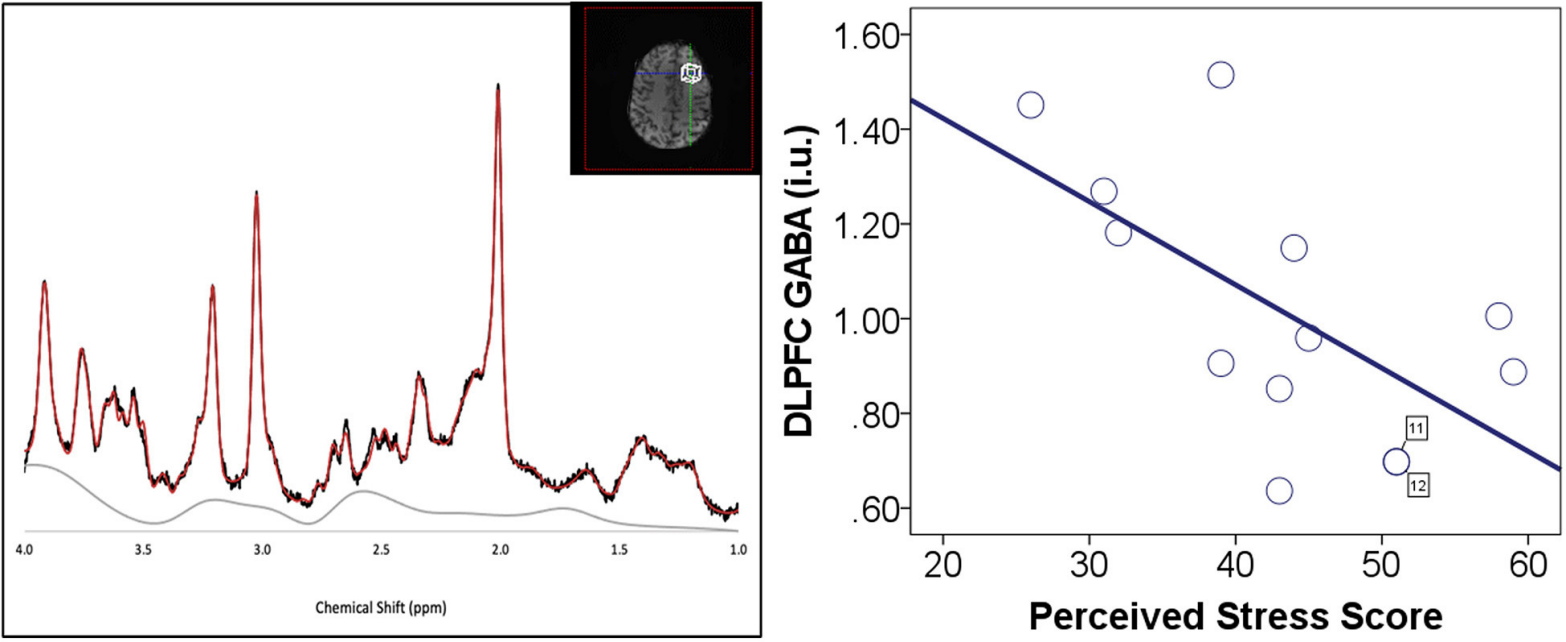
Perceived stress and DLPFC GABA. **(Left)** DLPFC voxel placement and example of spectrum. **(Right)** Negative correlation between perceived stress scale (PSS) scores and left DLPFC GABA. DLPFC GABA measurements are in institutional units. There were two participants (cases 11 and 12) with similar values for both DLPFC GABA and PSS score. These data points are labeled with the case number to identify the overlapping data points.

We observed a significant negative correlation between PSS scores and left DLPFC GABA (*r* = −0.62, *p* = 0.023; Table 1; Figure 1) while VMPFC GABA (*r* = −0.46, *p* = 0.086) and ACC GABA (*r* = 0.19, *p* = 0.479) were not significantly correlated with PSS scores (Supplementary Table 1).

### Perceived Stress and RSFC

Having observed a significant nominal correlation between left DLPFC GABA and PSS scores, subject-specific left DLPFC voxel masks were implemented as the seed ROIs in the seed-based ROI-to-ROI analyses. We extracted the Fisher-transformed *Z*-scores for all significant connections and carried them forward to test for relationships with PSS scores and GABA. Significant connections with the left DLPFC voxel carried forward included: bilateral LPFC, bilateral PPC, bilateral LP, bilateral RPFC and the ACC (Supplementary Figures 2, 3; Supplementary Table 2). In exploratory analyses we also extracted the Fisher-transformed *z*-scores for non-significant connections and tested the relationships with PSS as all *a priori* ROIs were of interest.

No significant correlations emerged between PSS scores and any of the primary RSFC connections tested (all *p* > 0.148; Table 2). For exploratory RSFC analyses with PSS scores we observed a trend toward a negative association between PSS scores and RSFC from the left DLPFC voxel to the left hippocampus (*r* = −0.52, *p* = 0.068; Supplementary Table 4).

**Table 2:** Correlations of PSS scores and DLPFC GABA with significant RSFC from the DLPFC voxel seeds.

**Target ROIs (Network)**	**L/R**	**PSS Score (*n =* 14)**	**DLPFC GABA (*n =* 15)**
Lateral prefrontal cortex (CEN)	L	*r* = 0.43, *p* = 0.148	***r*** **=** **−0.67**, ***p*** **=** 0.006^*^
Lateral prefrontal cortex (CEN)	R	*r* = 0.11, *p* = 0.722	*r* = −0.38, *p* = 0.158
Posterior parietal cortex (CEN)	L	*r* = 0.25, *p* = 0.408	*r* = −0.21, *p* = 0.459
Posterior parietal cortex (CEN)	R	*r* = 0.15, *p* = 0.616	*r* = −0.26, *p* = 0.354
Rostrolateral prefrontal cortex (SN)	L	*r* = −0.24, *p* = 0.426	*r* = 0.02, *p* = 0.951
Lateral parietal cortex (DMN)	L	*r* = 0.37, *p* = 0.214	*r* = −0.35, *p* = 0.198
Anterior cingulate cortex (SN)	L/R	*r* = −0.23, *p* = 0.448	*r* = 0.26, *p* = 0.246
Lateral parietal cortex (DMN)	R	*r* = 0.26, *p* = 0.390	*r* = −0.18, *p* = 0.521
Rostrolateral prefrontal cortex (SN)	R	*r* = −0.39, *p* = 0.192	*r* = 0.07, *p* = 0.801

* *Correlation is significant at the 0.05 level (2-tailed). Bold values indicate statistically significant results at a significance threshold of p < 0.05*.

### RSFC and DLPFC GABA

As expected, We observed a significant negative correlation between left DLPFC GABA with FC between our left DLPFC voxel and the left LPFC ROI from the Harvard-Oxford atlas in CONN, (*r* = −0.67, *p* = 0.006; Table 2). No significant associations were observed in the main analyses, or in exploratory analyses testing DLPFC GABA and nonsignificant RSFC (all *p* > 0.074; Supplementary Table 4). However, a trend in positive association emerged between left DLPFC GABA with left DLPFC voxel-MPFC FC (*r* = 0.47, *p* = 0.077), and a trend in negative association emerged between left DLPFC GABA and FC between the left DLPFC voxel and the left SMG, a node of the SN (*r* = −0.47, *p* = 0.074; Supplementary Table 4). Given the small sample size, formal tests of mediation and moderation were not feasible so we restricted our analyses to testing correlations between GABA and RSFC.

## Discussion

In this preliminary study we examined GABAergic function as a potential mechanism related to perceived stress and stress-related functional network dysregulation. We tested the association of self-reported perceived stress with brain GABA levels in prefrontal control regions along with corresponding alterations in RSFC in intrinsic networks and limbic regions implicated in the regulation of the stress response. We found that measures of perceived stress were negatively related to GABA levels in the left DLPFC. However, we did not observe a significant relationship between perceived stress and RSFC.

The DLPFC is thought to play an inhibitory role in the physiological response to stress via regulation of amygdala projections to hypothalamus, a key region of the HPA-axis known to be modulated by GABAergic activity in response to stress (23, 51, 52). The DLPFC has been targeted in neurostimulation treatment aimed at improving stress-related cognitive impairments and physiological responses. DLPFC stimulation by repetitive transcranial magnetic stimulation (rTMS) and transcranial direct current stimulation (tDCS) has been shown to increase GABA levels in the DLPFC and striatum, respectively (53, 54). Moreover, tDCS of the DLPFC has been shown to prevent acute stress-induced working memory deficits (55) and high frequency repetitive transcranial magnetic stimulation (HF-rTMS) over the left DLPFC applied before a social stress task blunted cortisol response (52). Our result showing an inverse relationship between perceived stress and left DLPFC GABA levels is consistent with these findings. However, we did not observe a relationship between PSS scores and GABA levels in the VMPFC or ACC, despite evidence implicating altered GABA levels in these regions in the etiology of chronic stress-related conditions such as major depressive disorder (MDD) and post-traumatic stress disorder (PTSD) (56).

Task-based functional neuroimaging studies suggest the left DLPFC and hippocampus along with the ventrolateral prefrontal cortex (VLPFC), dorsal ACC, and left insula make up a stress adaptation response network (57). RSFC studies have shown that FC between the hippocampus and the DLPFC increases in response to a stressor and predict feeling less subjective stress and lower subjective arousal in response to a stressor (58). In addition, decreased hippocampal FC to the precuneus and increase FC to the middle frontal gyrus are associated with greater perceived stress. Our finding of a trend in negative association between perceived stress and RSFC from the DLPFC voxel to the left hippocampus extends prior work indicating that increased FC between these regions predicts lower subject arousal to and feelings of stress and suggests a possible role for DLPFC-hippocampus FC in the stress adaption response.

Although we found perceived stress to be related to GABA levels in the left DLPFC but not to RSFC with the left DLPFC voxel seed, we did observe a significant negative relationship between GABA levels in the left DLPFC and RSFC between the left DLPFC voxel and the left LPFC node of the CEN. The LPFC includes the DLPFC, the ventrolateral prefrontal cortex (VLPFC) and frontal eye fields (59) and GABA+ levels in the DLPFC and VLPFC have been shown to be positively correlated (60); thus, it would be expected to find an association of left DLPFC GABA with connectivity between the left DLPFC and the left LPFC node. Previous research has shown that GABA concentrations in the mPFC are associated with between-network anticorrelations of the DMN and CEN but that DLPFC GABA concentrations did not play a significant role in between-network interactions (61). In contrast, our result of a trend in positive association between DLPFC GABA and DLPFC voxel-MPFC FC implicates DLPFC GABA in between-network interactions of the CEN and DMN but in terms of increased connectivity rather than anticorrelation between the DLPFC and MPFC nodes. Additionally, the CEN is typically more active during cognitive tasks requiring externally directed thought (19) and anti-correlated with the DMN at rest (16). We also observed a trend in negative association between DLPFC GABA levels and FC from DLPFC voxel to left SMG, a node in the SN. Functional imaging studies have revealed decreased DLPFC activity and increased SMG activity in response to stress induction (62) and increased SMG activity has also been shown to correlate with stress-induced cortisol measures (63). Taken together these results trends suggest that it is possible that the association of DLPFC GABA levels with PSS may be related to aberrant CEN-DMN and CEN-SN interactions. However, studies involving larger samples sizes are need to better elucidate these results.

Findings of the current study should be evaluated with consideration of several limitations. The sample size in this study is small, limiting our ability to detect small effects and to test for effects of potential confounding, mediating and moderating variables. However, this preliminary study aimed to generate hypotheses to be tested in future studies and provides valuable initial data related to the role of GABAergic functioning in perceived stress that warrant further investigation. This study did not include biological measurements of HPA-axis activity or autonomic nervous system function response to stress. The collection of such measures would capture additional facets underlying stress and the perception of stress that may additionally be related to GABAergic functioning and RSFC. Given the large voxel sizes utilized in MRS, biologically important heterogeneity within each voxel may not be captured. For rsfMRI in this preliminary study, we implemented a high-pass filter only in the pre-processing of the data. Though others have recommended this decision the use of band-pass filtering instead is still debated in rsfMRI research. We therefore note that our analyses may be less generalizable to studies using band-pass filtering in the processing pipeline. In addition, we limited our analyses to *a priori* atlas-defined ROI and thus do not report on whole-brain seed-to-voxel analyses that might have identified additional regions that are biologically relevant to perceived stress. Such analyses would be of interest in future studies in larger samples, as would formal statistical examination of GABA as a potential modifier or mediator of the relationship between perceived stress and RSFC together with the impact of diagnosis, age, sex, and biological measurements of HPA-axis activity, all of which were beyond the scope of this preliminary study.

## Conclusions

In the present study, we conducted a preliminary investigation regarding the relationship of perceived stress, brain GABA levels, and RSFC in stress-related networks and limbic regions. Our preliminary results support an association between perceived stress and dysregulated GABAergic functioning in DLPFC, a core node of the CEN, an intrinsic network thought to underlie goal-directed attentional processes. This line of work has potential to shed light on GABAergic and other neurobiological mechanisms that may contribute to increased perception of stress experiences and coping which may in turn aid in the development of prevention and treatment strategies to improve outcomes in stress-related conditions.

## Data Availability Statement

The original contributions presented in the study are included in the article/Supplementary Material, further inquiries can be directed to the corresponding author/s.

## Ethics Statement

The study protocol was reviewed and approved by the Mass General Brigham Human Subjects Committee. The patients/participants provided their written informed consent to participate in this study.

## Author Contributions

PM, AL, JT, and LH contributed to the study design. EC, WZ, and JB performed data analysis. HL and WZ contributed to data collection. All authors contributed to manuscript preparation.

## Funding

This work was supported by grants from the Brigham and Women's Hospital Connors Center for Women's Health and Gender Biology WHISPR Pilot Award (PM) and from the National Institutes of Health R01MH110979 (PM), U54AG062322 (PM) and T32MH017119 (JB).

## Conflict of Interest

The authors declare that the research was conducted in the absence of any commercial or financial relationships that could be construed as a potential conflict of interest.

## Publisher's Note

All claims expressed in this article are solely those of the authors and do not necessarily represent those of their affiliated organizations, or those of the publisher, the editors and the reviewers. Any product that may be evaluated in this article, or claim that may be made by its manufacturer, is not guaranteed or endorsed by the publisher.
