# Erratum: Perceived Stress, Cortical GABA, and Functional Connectivity Correlates: A Hypothesis-Generating Preliminary Study

**DOI:** 10.3389/fpsyt.2022.902707

**Published:** 2022-04-20

**Authors:** Frontiers Production Office

**Affiliations:** Frontiers Media SA, Lausanne, Switzerland

**Keywords:** perceived stress, gamma-aminobutyric acid, magnetic resonance spectroscopy, prefrontal cortex, rfMRI

Due to a production error, the wrong supplementary figures were published, and an incorrect file was published.

New files “Supplementary Image 1,” “Supplementary Image 2,” and “Supplementary Image 3” have been published. “Supplementary Image 4” has been unpublished.

The publisher apologizes for these mistakes. The original version of this article has been updated.

