# Supplementary material for: Perceived Stress, Cortical GABA, and Functional Connectivity Correlates: A Hypothesis-Generating Preliminary Study
Source: Front Psychiatry. 2022 Mar 8;13:802449. doi: 10.3389/fpsyt.2022.802449 (PMC8957825; doi:10.3389/fpsyt.2022.802449)
Supplement: Supplementary file 1 [file Table_1.docx]

| **Supplementary Table 1. Demographic, clinical characteristics, and relevant relationships with PSS Scores and ACC GABA and VMPFC GABA** | | | |
| --- | --- | --- | --- |
|  | n=17 | **ACC GABA (n=16)** | **VMPFC GABA (n=16)** |
| Age | 47.2 (8.2) | *r* = -0.14, *p* = 0.607 | *r* = 0.16, *p* = 0.556 |
| Sex (% female) | 70.6% | *t*_14_  = 0.29, *p* = 0.780 | *t*_14_ = -0.72, *p* = 0.479 |
| Handedness (% RH) | 76.5% | *t*_14_ = 1.00, *p* = 0.332 | *t*_14_ = 0.76, *p* = 0.458 |
| Mood Diagnosis (% yes) | 47.1% | *t*_14_  = 0.60, *p* = 0.557 | *t*_14_ = -0.77, *p* = 0.456 |
| Trauma Experience (% yes) | 35.3% | *t*_14_ = 1.17, *p* = 0.262 | *t*_14_ = -1.32, *p* = 0.208 |
| MADRS | 2.3 (6.0) | *r* = -0.02, *p* = 0.945 | *r* = -0.13, *p* = 0.652 |
| YMRS | 1.7 (2.5) | *r* = -0.10, *p* = 0.709 | *r* = -0.29, *p* = 0.297 |
| PSS | 43.2 (9.6) | *r* = 0.19, *p* = 0.524 | *r* = -0.47, *p* = 0.107 |
| ACC GABA (i.u.) | 1.5 (0.8) | N/A | *r* = -0.38, *p* = 0.165 |
| VMPFC GABA (i.u.) | 1.8 (0.7) | *r* = -0.38, *p* = 0.165 | N/A |
| Results presented as mean(SD) or %. PSS=Perceived Stress Scale, MADRS=Montgomery-Asberg Depression Rating Scale, YMRS=Young Mania Rating Scale, RH=Right Hand, STAI-S=State-Trait Anxiety Inventory-State. ACC GABA= Anterior Cingulate Cortex Gamma-Amino Butyric Acid, VMPFC=VentroMedial PreFrontal Cortex. i.u.=institutional units. For dichotomous variables the group shown represents the comparison group and the group not shown represents the reference group for statistical testing. | | | |
