# Supplementary material for: Perceived Stress, Cortical GABA, and Functional Connectivity Correlates: A Hypothesis-Generating Preliminary Study
Source: Front Psychiatry. 2022 Mar 8;13:802449. doi: 10.3389/fpsyt.2022.802449 (PMC8957825; doi:10.3389/fpsyt.2022.802449)
Supplement: Supplementary file 2 [file Table_2.docx]

| **Supplementary Table 2. Functional Connectivity from Subject-Specific DLPFC Voxels seeds to DMN, CEN, SN, and Limbic Regions (n=16)** | | | | | |
| --- | --- | --- | --- | --- | --- |
| **Target ROI (Network)** | **L/R** | ***β*** | ***T*(15)** | ***p*-unc** | ***p*-FDR** |
| Lateral Prefrontal Cortex (CEN) | L | 0.49 | 7.61 | <0.001 | **<0.001**** |
| Lateral Prefrontal Cortex (CEN) | R | 0.36 | 7.03 | <0.001 | **<0.001**** |
| Posterior Parietal Cortex (CEN) | L | 0.39 | 5.86 | <0.001 | **<0.001**** |
| Posterior Parietal Cortex (CEN) | R | 0.29 | 4.51 | <0.001 | **0.002**** |
| Rostrolateral Prefrontal Cortex (SN) | L | 0.33 | 4.30 | 0.001 | **0.002**** |
| Lateral Parietal Cortex (DMN) | L | 0.27 | 4.04 | 0.001 | **0.003**** |
| Anterior Cingulate Cortex (SN) | L/R | 0.29 | 3.81 | 0.002 | **0.005**** |
| Lateral Parietal Cortex (DMN) | R | 0.21 | 3.45 | 0.004 | **0.009**** |
| Rostrolateral Prefrontal Cortex (SN) | R | 0.17 | 2.58 | 0.021 | **0.045*** |
| Medial Prefrontal Cortex (DMN) | L/R | 0.11 | 1.95 | 0.070 | 0.132 |
| Anterior Insula (SN) | L | 0.09 | 1.48 | 0.158 | 0.274 |
| Hippocampus | R | -0.05 | -1.40 | 0.183 | 0.289 |
| Posterior Cingulate Cortex (DMN) | L/R | 0.06 | 1.27 | 0.223 | 0.326 |
| Hippocampus | L | -0.02 | -0.79 | 0.445 | 0.603 |
| Anterior Insula (SN) | R | 0.04 | 0.66 | 0.520 | 0.658 |
| Amygdala | R | 0.01 | 0.51 | 0.615 | 0.730 |
| Amygdala | L | 0.01 | 0.35 | 0.731 | 0.817 |
| Supramarginal Gyrus (SN) | L | 0.01 | 0.26 | 0.798 | 0.843 |
| ***** Significant at the 0.05 level.  ****** Significant at the 0.01 level. | | | | | |
