# Supplementary material for: Perceived Stress, Cortical GABA, and Functional Connectivity Correlates: A Hypothesis-Generating Preliminary Study
Source: Front Psychiatry. 2022 Mar 8;13:802449. doi: 10.3389/fpsyt.2022.802449 (PMC8957825; doi:10.3389/fpsyt.2022.802449)
Supplement: Supplementary file 3 [file Table_3.docx]

| **Supplementary Table 3. MRS Quality Control Measures** | | | |
| --- | --- | --- | --- |
| **Voxel Location** | **FWHM (Hz)** | **SNR** | **GABA CRLB (%)** |
| DLPFC  ACC  VMPFC | 18.3 (4.3)  17.4 (3.9)  18.4 (4.1) | 32.1 (10.5)  50.8 (11.0)  34.2 (11.3) | 12.2 (3.3)  6.6 (2.1)  9.4 (3.5) |
