# Supplementary material for: Perceived Stress, Cortical GABA, and Functional Connectivity Correlates: A Hypothesis-Generating Preliminary Study
Source: Front Psychiatry. 2022 Mar 8;13:802449. doi: 10.3389/fpsyt.2022.802449 (PMC8957825; doi:10.3389/fpsyt.2022.802449)
Supplement: Supplementary file 4 [file Table_4.docx]

| **Supplementary Table 4.  Correlations of PSS Scores and DLPFC GABA with Non-Significant RSFC from the DLPFC Voxel Seeds** | | | |
| --- | --- | --- | --- |
| **Target ROIs (Network)** | **L/R** | **PSS Score (n=14)** | **DLPFC GABA (n=15)** |
| Medial Prefrontal Cortex (DMN) | L/R | *r* = -0.28, *p* = 0.354 | *r* = 0.47, *p* = 0.077 |
| Anterior Insula (SN) | L | *r* = 0.04, *p* = 0.907 | *r* = -0.22, *p* = 0.440 |
| Hippocampus | R | *r* = -0.26, *p* = 0.401 | *r* = 0.28, *p* = 0.306 |
| Posterior Cingulate Cortex (DMN) | L/R | *r* = 0.02, *p* = 0.940 | *r* = -0.17, *p* = 0.543 |
| Hippocampus | L | *r* = -0.52, *p* = 0.068 | *r* = 0.33, *p* = 0.232 |
| Anterior Insula (SN) | R | *r* = -0.13, *p* = 0.680 | *r* = -0.24, *p* = 0.392 |
| Amygdala | R | *r* = -0.20, *p* = 0.516 | *r* = 0.23, *p* = 0.407 |
| Amygdala | L | *r* = -0.09, *p* = 0.776 | *r* = -0.31, *p* = 0.266 |
| Supramarginal Gyrus (SN) | L | *r* = -0.22, *p* = 0.469 | *r* = -0.47, *p* = 0.074 |
| ***** Correlation is significant at the 0.05 level (2-tailed). | | | |
