# Supplementary material for: Perceived Stress, Cortical GABA, and Functional Connectivity Correlates: A Hypothesis-Generating Preliminary Study
Source: Front Psychiatry. 2022 Mar 8;13:802449. doi: 10.3389/fpsyt.2022.802449 (PMC8957825; doi:10.3389/fpsyt.2022.802449)

Supplementary Figure 1. Voxel placement examples and corresponding spectra. Representative voxel placement examples with corresponding spectra for the anterior cingulate cortex (ACC; top), ventromedial prefrontal cortex (VMPFC; middle), and the dorsolateral prefrontal cortex (DLPFC; bottom).

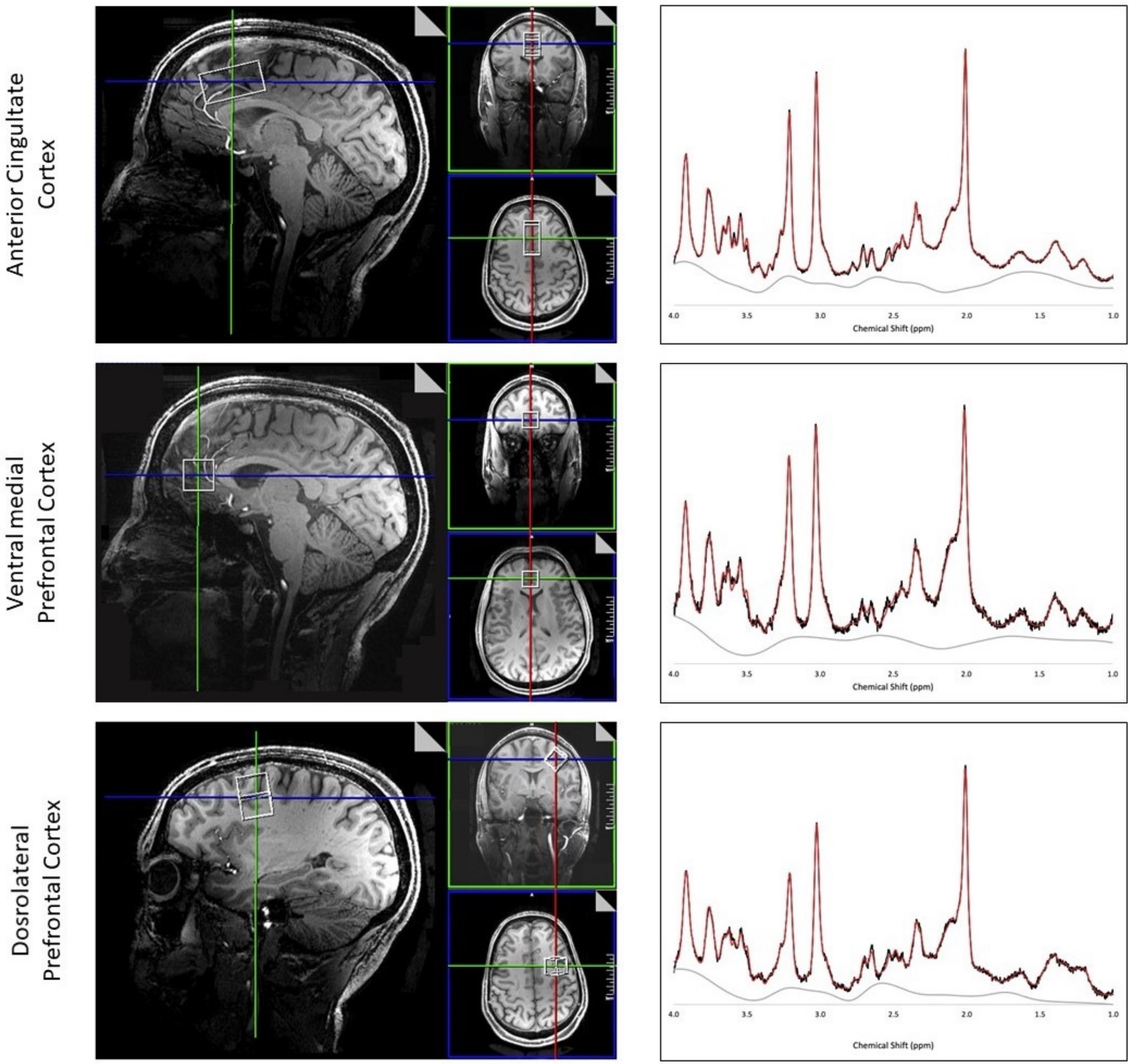

Supplement: Supplementary file 5 [file Image_1.pdf]
