# Supplementary material for: Perceived Stress, Cortical GABA, and Functional Connectivity Correlates: A Hypothesis-Generating Preliminary Study
Source: Front Psychiatry. 2022 Mar 8;13:802449. doi: 10.3389/fpsyt.2022.802449 (PMC8957825; doi:10.3389/fpsyt.2022.802449)

Supplementary Figure 2. Significant resting state connections from the left LPFC. Derived from ROI-to-ROI analyses in the whole sample using the left LPFC as seed region to target regions in the default mode network (DMN), salience network (SN), central executive network (CEN) and limbic regions (amygdala and hippocampus). Shown are connections with voxel-wise threshold of $p_{\text{uncorrected}} < 0.001$ and cluster-level threshold of $p_{FDR} < 0.05$. **(A)** Glass brain showing significant connections from the left LPFC to CEN nodes (right LPFC, bilateral PPC), DMN nodes (bilateral LP), SN nodes (bilateral RPFC), and limbic region (right amygdala). Red indicates positive correlation. Blue indicates anticorrelation. **(B)** Bar graph representing average correlation coefficients for each significant connection with error bars representing standard error of the mean correlation. LPFC = lateral prefrontal cortex, PPC = posterior parietal cortex, LP = lateral parietal, RPFC = rostral prefrontal cortex.

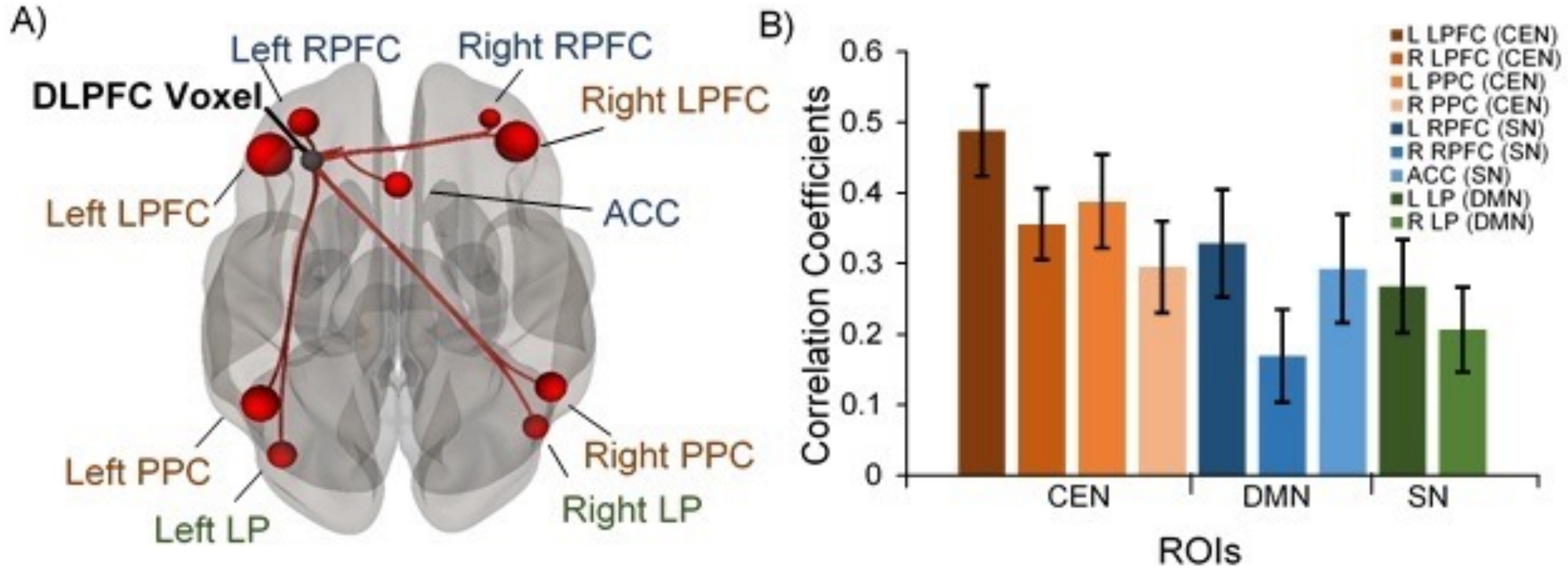

Supplement: Supplementary file 6 [file Image_2.pdf]
