# Supplementary material for: Perceived Stress, Cortical GABA, and Functional Connectivity Correlates: A Hypothesis-Generating Preliminary Study
Source: Front Psychiatry. 2022 Mar 8;13:802449. doi: 10.3389/fpsyt.2022.802449 (PMC8957825; doi:10.3389/fpsyt.2022.802449)

Supplementary Figure 3. Heatmap of DLPFC voxel placement. Heatmap showing the overlap of subject-specific DLPFC voxel placements. Color bar represents consistency of overlap. Placement of voxel was optimized for individual anatomy.

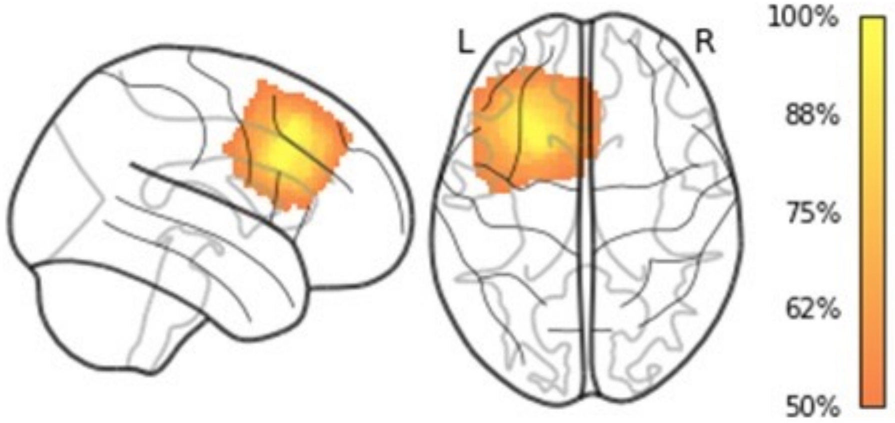

Supplement: Supplementary file 7 [file Image_3.pdf]
